# Cardiopulmonary Exercise Testing Reveals Functional Limitations and Work Disability in Severe Post-COVID-19 and ME/CFS Patients

**DOI:** 10.1186/s40798-026-00995-1

**Published:** 2026-04-27

**Authors:** Aleksandar Tomaskovic, Vincent Weber, David T. Ochmann, Barlo Hillen, Elmo W. I. Neuberger, Alexandra Brahmer, Ella Lachtermann, Klaus Lieb, Perikles Simon

**Affiliations:** 1 Department of Sports Medicine, Prevention and Rehabilitation, Institute for Sport Science, Johannes Gutenberg-University Mainz, Mainz, Germany; 2 Clinic of Psychiatry and Psychotherapy, University Medical Center, Johannes Gutenberg-University Mainz, Mainz, Germany; 3 Institute for Occupational, Social and Environmental Medicine, University Medical Center, Johannes Gutenberg-University Mainz, Mainz, Germany

**Keywords:** Long COVID, Exercise physiology, Occupational medicine, Functional work capacity, Rehabilitation

## Abstract

**Background:**

Patients severely affected by post-COVID-19 condition (PCC) and myalgic encephalomyelitis/chronic fatigue syndrome (ME/CFS) often experience long-term work incapacity, contributing to a growing economic burden. Organ-centered clinical diagnostics frequently fail to explain their work disability.

**Objectives:**

We aimed to objectively assess physical work ability using cardiopulmonary exercise testing (CPET) in a cohort of long-standing and severely affected PCC patients. We hypothesized: (1) patients with ME/CFS exhibit lower peak oxygen uptake (VO₂_peak_ [mL/min/kg]) and peak power output (PPO [W/kg]) than those without; (2) most patients demonstrate objective work disability, closely aligned with subjective perception of disability; (3) oxygen pulse (O_2_ pulse [mL/bpm]) is reduced in ME/CFS, independent of comorbidity.

**Methods:**

The study was conducted in the Department of Sports Medicine, Prevention and Rehabilitation at Johannes Gutenberg-University Mainz (Mainz, Germany). Between July 31, 2023, and March 31, 2025, a total of 92 PCC patients with suspected occupational disease underwent symptom-limited CPET and completed the Canadian Consensus Criteria, Bell Disability Scale (Bell-Score), and DePaul Symptom Questionnaire (Post-Exertional Malaise) Short Form (DSQ-PEM).

**Results:**

Nearly half of the patients (49%) met ME/CFS criteria and 79% screened positive on the DSQ-PEM. ME/CFS patients showed significantly lower VO₂_peak_ (13.0 ± 3.1 vs. 15.4 ± 4.9, p = 0.012), PPO (0.9 ± 0.3 vs. 1.1 ± 0.5, p = 0.014), and O₂ pulse (7.7 ± 2.0 vs. 8.5 ± 1.9, p = 0.047) compared to those without ME/CFS. Overall, 66% of patients met objective thresholds for work disability (VO₂_peak_ < 15 mL/min/kg or PPO < 1 W/kg). Forty-five patients (51%) had a Bell-Score ≤ 30 and 82% from those had VO₂_peak_ < 15 and/or PPO < 1. VO₂_peak_ and PPO significantly correlated with Bell-Score (r = 0.3, p = 0.005 and r = 0.3, p = 0.003) and were the lowest among patients on medical sick leave (13.3 ± 3.3 and 0.9 ± 0.3), compared to those in occupational reintegration (16.0 ± 3.9, p = 0.04 and 1.2 ± 0.5, p = 0.024) or currently working (18.0 ± 7.1, p = 0.036 and 1.2 ± 0.5, p = 0.015).

**Conclusions:**

Severely affected PCC patients exhibit objective work disability, particularly those with ME/CFS. VO₂_peak_ and PPO are associated with subjective disability and occupational status. Therefore, early integration of CPET into clinical and occupational evaluations can inform individualized therapy planning and return-to-work decisions.

*Trial registration* DRKS, DRKS00032394. Registered 28 July 2023, https://drks.de/search/de/trial/DRKS00032394

**Supplementary Information:**

The online version contains supplementary material available at 10.1186/s40798-026-00995-1.

## Key Points

- Severe post-COVID-19 condition patients, particularly those meeting myalgic encephalomyelitis/chronic fatigue syndrome criteria, exhibit markedly reduced peak oxygen uptake (VO₂_peak_ < 15 mL/min/kg) and peak power output (PPO < 1 W/kg), reflecting absolute work disability.
- Submaximal cardiopulmonary exercise testing parameters (e.g., VO₂ at first ventilatory threshold < 11 mL/min/kg or < 40% predicted VO₂) can detect severe functional limitations in patients unable to reach maximal effort.
- Reductions in VO₂_peak_ and PPO are largely independent of comorbidities, supporting functional rather than structural limitations as the primary mechanism.
- VO₂_peak_ and PPO are associated with subjective disability (Bell Disability Scale) and occupational status, highlighting the use of cardiopulmonary exercise testing for clinical assessment, rehabilitation planning, and return-to-work decisions.

**Supplementary Information:**

The online version contains supplementary material available at 10.1186/s40798-026-00995-1.

## Introduction

The COVID-19 pandemic has affected over 775 million people worldwide [1]. While the majority recover within weeks, approximately 10–20% develop persistent symptoms lasting beyond 12 weeks, a condition referred to as post-COVID-19 condition (PCC) [2]. PCC encompasses a wide range of symptoms: fatigue, cognitive deficits and physical impairments that persist beyond 12 weeks post-infection and impair quality of life and work ability [3–7]. Within the PCC population, 9.8%–15.4% fulfill the Canadian Consensus Criteria (CCC) [8] for myalgic encephalomyelitis/chronic fatigue syndrome (ME/CFS) [9–11]. ME/CFS is defined by chronic fatigue lasting for more than 6 months and the presence of post-exertional malaise (PEM). PEM refers to a disproportionate worsening of symptoms following physical, cognitive or mental exertion, often delayed by up to 72 h after the triggering activity and without a clear dose–response relationship [12, 13].

The etiology of PCC and ME/CFS is incompletely understood and is considered multifactorial, involving interacting biological mechanisms across immune, neurological, vascular, and metabolic systems [14, 15]. Despite growing pathophysiological insights, the diagnosis of PCC and ME/CFS primarily relies on patient-reported outcome measures (PROMs) and the exclusion of alternative medical conditions [16]. The subjective approach, referring to symptom-based clinical assessment relying primarily on patient self-report and clinician judgement, presents substantial challenges, particularly in patients with pronounced fatigue or cognitive dysfunction, where symptom reporting may be unreliable and physician decision-making affected [8]. In addition, the exclusion process often depends on organ-centered diagnostics, which may yield unremarkable cardiovascular, pulmonary, or neurological findings. This disconnect is especially problematic when evaluating work ability, as isolated, organ or system-specific assessments offer limited insight into global functional impairment.

Alongside qualitative assessments of clinical symptoms and functional impairment using PROMs [8, 16, 17] and organ-centered clinical diagnostics, cardiopulmonary exercise testing (CPET) provides objective data on acute cardiopulmonary exercise response [18, 19] and physical work ability [20–23]. In PCC, CPET may also effectively complement qualitative assessments by identifying exertional limitations [24], distinguishing deconditioning [25, 26] from pathophysiological dysfunction [27] and likely objectively evaluating physical work ability. However, previous CPET studies mainly explored impaired physical capacity in relation to pathophysiological mechanisms of PCC [26]. To date, no study has systematically examined the association between cardiopulmonary exercise response and objectively assessed physical capacity in relation to work ability, particularly in individuals with long-standing and severe PCC. Given the rising prevalence of unexplained functional impairments in this population, and the urgent need for objective assessment of work ability, we conducted the study to address this gap. The study integrated PROMs, clinical assessments and symptom-limited CPET to comprehensively assess the physical work ability of individuals with long-standing and severe PCC.

We hypothesized that PCC patients who meet the diagnostic criteria for ME/CFS would exhibit significantly lower physical capacity, defined by lower VO₂_peak_ [mL/min/kg body mass] and peak power output (PPO [W/kg body mass]), compared to those without ME/CFS. As a secondary hypothesis, we proposed that most PCC patients would demonstrate physical work disability, defined by a VO₂_peak_ < 15 mL/min/kg or PPO < 1 W/kg [21, 23]. Furthermore, we expected that objectively measured physical capacity, would significantly correlate with subjectively reported functional disability, as measured by the Bell Disability Scale (Bell-Score) [17], and would vary according to occupational status. Finally, we proposed that CPET may non-invasively help identify peripheral mechanisms of exercise intolerance—specifically, impaired systemic oxygen extraction [24, 28] through the analysis of oxygen pulse (O₂ pulse [mL/bpm]).

## Methods

### Study Design

This cross-sectional observational study reports baseline clinical and exercise physiology data from the randomized controlled trial “Multimodal Web-Based Telerehabilitation for Patients with Post-COVID-19 Condition” [29]. The study was conducted in collaboration with the German Social Accident Insurance for Non-Governmental Health and Social Institutions (German: Berufsgenossenschaft für Gesundheitsdienst und Wohlfahrtspflege).

Ethical approval was obtained from the Rhineland-Palatinate Medical Association Ethics Committee (Mainz, 06/2023; Reg. No.: 2023–17082). All procedures complied with the Declaration of Helsinki, and written informed consent was obtained from the participants. The trial was prospectively registered in the German Clinical Trials Register on July 28, 2023 (DRKS00032394).

### Experimental Setup

**Fig. 1:**
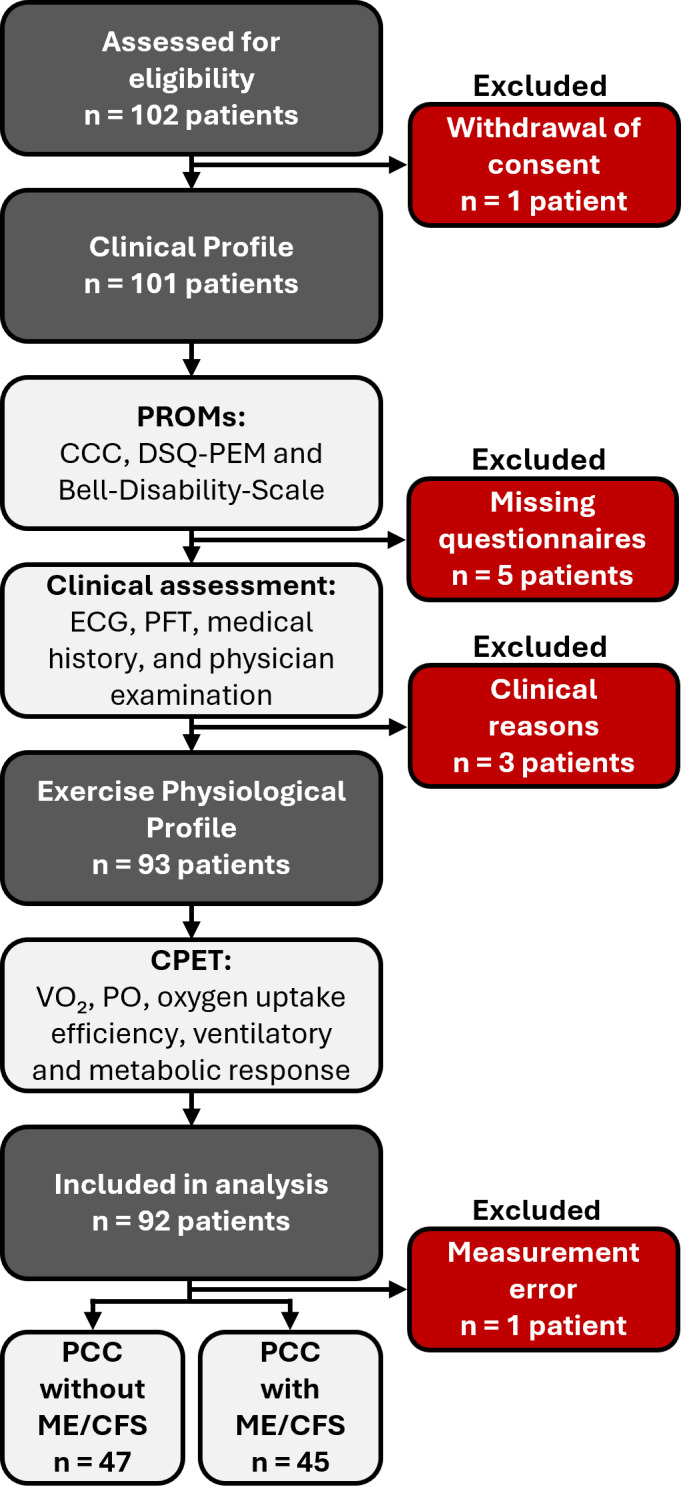
Study Flowchart of patient screening, exclusions, assessments, and final analytic sample. Analyses were performed for the total cohort (n = 92) and in subgroups with and without ME/CFS. *PCC* post-COVID-19 condition; *ME/CFS* myalgic encephalomyelitis/chronic fatigue syndrome, *PROMs* patient-reported outcome measures, *CCC* Canadian Consensus Criteria for ME/CFS, *DSQ-PEM* DePaul Symptom Questionnaire (Post-Exertional Malaise) Short Form, *ECG* electrocardiogram, *PFT* pulmonary function test, *CPET* cardiopulmonary exercise testing, *VO*_2_ oxygen uptake, *PO* power output

Between July 31, 2023, and March 31, 2025, a total of 102 patients with PCC were screened for eligibility (Fig. 1). One patient withdrew consent, leaving 101 patients who underwent initial clinical characterization, including standardized PROMs. Five patients were excluded due to incomplete or missing questionnaire data. The remaining 96 patients proceeded to further clinical characterization, comprising electrocardiography (ECG), pulmonary function testing, medical history, and physician examination. Three patients were unable to undergo CPET due to clinical contraindications: one due to orthopedic limitation and two due to disease-related muscle weakness. This resulted in a final sample of 93 patients undergoing exercise physiological characterization.

All 93 patients underwent CPET, but one dataset was excluded because a leaking respiratory mask caused a missing respiratory response, resulting in a final analytical sample of 92 patients. Data were analyzed for the entire cohort and stratified by ME/CFS status, comparing patients meeting the CCC criteria for ME/CFS (n = 45) with those who did not (n = 47).

### Study Participants

Participants were recruited through the Department of Sports Medicine, Prevention and Rehabilitation at Johannes Gutenberg-University Mainz (Mainz, Germany). Eligibility was determined according to predefined inclusion and exclusion criteria (Table 1).

**Table 1:** Inclusion and exclusion criteria

Inclusion criteria
1. Age ≥ 18 years
2. Confirmed SARS-CoV-2 infection by PCR performed by medical personnel
3. PCC diagnosis established by a family or occupational physician (main diagnosis + ICD-10: U09.9)
4. Temporary or complete occupational disability due to PCC
5. Study participants are insured by the BGW
Exclusion criteria
1. Presence of red flags [31] or absolute contraindications for moderate-to-vigorous physical activities [18]
2. Participation in another study
3. Inability to perform cycle ergometer testing

*PCR* Polymerase chain reaction,* ICD-10* International classification of diseases,* BGW* German Social Accident Insurance for Non-Governmental Health and Social Institutions,* PCC* post-COVID-19 condition

The study population consisted of employees from the German healthcare and social services sectors who were unable to work full- or part-time due to PCC. All individuals had documented occupational SARS-CoV-2 infection and persistent PCC symptoms for more than 12 weeks [30].

At enrollment, they were either on long-term medical leave or working reduced hours due to functional limitations. All had initiated procedures for official recognition of PCC as an occupational disease through the German Social Accident Insurance for Non-Governmental Health and Social Institutions.

### Outcome Measures

A comprehensive overview of all assessments and outcomes included in the “Multimodal Web-Based Telerehabilitation for Patients with Post-COVID-19 Condition” study is provided in the published study protocol [29]. The present manuscript focuses on PROMs, clinical assessments, and CPET data obtained during the baseline examination.

#### Patient-Reported Outcome Measures

Prior to their visit to the Sports Medicine Outpatient Clinic at Johannes Gutenberg University Mainz, participants completed three validated clinical questionnaires within a 7-day period, following a structured, stepwise procedure.

Two instruments, established for the diagnosis of ME/CFS and evaluation of functional impairment, were applied: the CCC [8], a clinical case definition for ME/CFS, and the Bell Disability Scale [17]. The diagnosis of ME/CFS according to the CCC requires a minimum duration of illness of six months. Fatigue and PEM must be present, as well as at least one symptom from the areas of sleep disturbance or pain, and at least two neurological/cognitive symptoms and manifestations from at least two of the autonomic, neuroendocrine, or immunological categories.

The DePaul Symptom Questionnaire (Post-Exertional Malaise) Short Form (DSQ-PEM) [16] was employed to specifically assess PEM.

#### Clinical Assessments

All participants underwent comprehensive clinical assessment prior to CPET, which included anthropometric measurements (body mass, body height, and body mass index [kg/m^2^]). A 12-lead ECG (CardioPart 12 PC, AMEDTEC Medizintechnik GmbH, Aue-Bad Schlema, Germany) was performed, along with blood pressure measurement using a digital sphygmomanometer (ERKA, Bad Tölz, Germany). Pulmonary function testing was conducted using body plethysmography (Bodybox 5500, MEDISOFT GmbH, Hamburg, Germany) to measure forced vital capacity (FVC), forced expiratory volume in one second (FEV_1_) and peak expiratory flow (PEF).

Medical history and physical examination were conducted by a study physician (EL, internal medicine) following the IPPAF algorithm (inspection, percussion, palpation, auscultation, and functional assessment). Clinical data were independently reviewed by two physicians, with discrepancies resolved by consensus.

Based on medical history and examination, patients were categorized into two clinical subgroups: those with multimorbidity (defined as ≥ 3 comorbidities and/or ≥ 1 severe acute or chronic disease) and those without (defined as < 3 comorbidities and no severe disease) [32]. Severe disease was defined as a condition significantly impairing physical capacity, such as heart failure (New York Heart Association Stages ≥ III) [33] or advanced chronic obstructive pulmonary disease [34].

#### Cardiopulmonary Exercise Testing

CPET was used to objectively assess physical work ability. The analysis focused on CPET-derived parameters reflecting physical capacity, oxygen uptake efficiency, and ventilatory and metabolic responses.

**Fig. 2:**
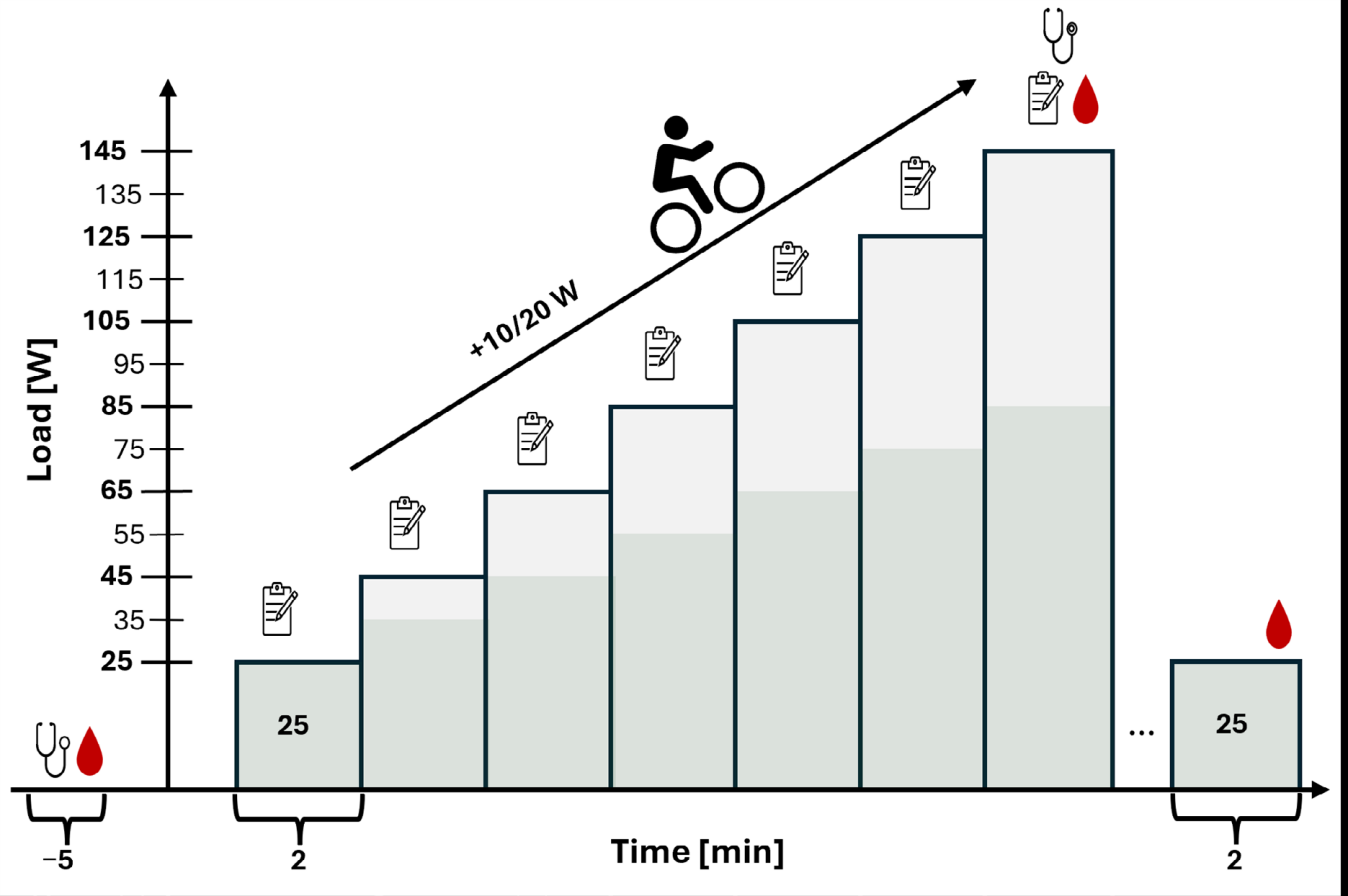
Exemplary CPET Protocols. Red filled blood drop: capillary blood collection (5 min before CPET, and immediately and three-minute after CPET); Stethoscope: blood pressure measurement; Notepad: Rating of perceived exertion (additionally, primary termination criteria only at the last stage). *CPET* cardiopulmonary exercise testing, *W* workload in watts

The CPET was conducted on a cycle ergometer (ER 900PC, Ergoline GmbH, Bitz, Germany) and was designed as a single maximal symptom-limited CPET, aiming for 4–6 stages of 2 min each, with incremental increases of 10 or 20 Watt [W] at the end of each stage (Fig. 2). The duration of the recovery phase after termination of the test is 3 min (1 min resting and 2 min with an external load of 25 W if possible).

The test duration was designed to last 8–12 min to optimize the assessment of VO_2peak_ and PPO [22, 35]. To minimize the risk of premature exhaustion, two individualized load progression protocols were applied. The protocol was selected based on self-reported fitness state and clinical assessment. Perceived exertion was assessed using the Borg scale (6–20) during the final 30 s of each stage [36]. CPET was terminated upon meeting absolute or relative termination criteria (Supplementary File 1). Primary termination criteria and subjective symptoms (e.g., fatigue, leg discomfort, angina, dyspnea) were recorded immediately after CPET completion.

Minute ventilation (VE), oxygen uptake (VO₂), and carbon dioxide production (VCO₂) were measured breath-by-breath using the ERGOSTIK CPET system and the BLUE CHERRY diagnostic software platform (Geratherm Respiratory, Bad Kissingen, Germany). Raw data were filtered with a 3rd-order low-pass forward–backward Butterworth filter (0.04 Hz cut-off, zero lag) to remove noise and artifacts. This method, implemented in the spiro package (R version 0.0.4) [37], is recommended by Nolte et al. [38].

VO₂_peak_ [mL/min/kg] was defined as the highest recorded oxygen uptake during CPET, and PPO [W] as the highest workload achieved. Further details on VO₂_peak_ determination are provided in Supplementary File 2. To ensure diagnostic validity, the analysis of physical work ability (3.4: Diagnostic Utility of CPET for Physical Work Ability) was conducted for the full cohort and a subgroup that met physiological exertion criteria defined by a respiratory exchange ratio (RER) ≥ 1.0. This threshold indicate substantial anaerobic contribution and is accepted as a marker of sufficient exertion in clinical populations with limited exercise tolerance [19, 22].

O₂ pulse [mL/bpm] was calculated as VO₂ divided by heart rate and serves as a non-invasive surrogate for systemic oxygen extraction, reflecting circulatory and muscular oxygen utilization capacity [39, 40]. Additionally, the oxygen uptake efficiency slope (OUES) was computed by plotting VO₂ [mL/min] against the logarithm of VE [L/min] during the exercise phase. OUES represented an integrated submaximal index of ventilatory and circulatory efficiency [19, 41].

The VE/VCO₂ slope, an indicator of ventilatory efficiency, was determined using linear regression of VE and VCO₂ during the entire exercise phase, as proposed by Ingle et al. [42]. In addition, ventilatory equivalents for oxygen (VE/VO₂) and carbon dioxide (VE/VCO₂) were calculated to assess the ventilatory response relative to gas exchange demands. End-tidal partial pressures of oxygen (PETO₂) and carbon dioxide (PETCO₂) were analyzed as indicators of gas exchange efficiency and ventilatory-perfusion matching [22].

First and second ventilatory thresholds (VT1 and VT2) were determined based on established gas exchange criteria (Supplementary File 3) [22] by two independent sports scientists. These thresholds served as indicators of submaximal physical capacity and metabolic transitions during exercise.

A 12-lead electrocardiogram (ECG) was continuously monitored throughout the test (CardioPart 12 PC; AMEDTEC Medizintechnik GmbH, Aue-Bad Schlema, Germany). The blood pressure was measured manually while the patient was sitting on the cycle ergometer in a resting condition and immediately after the exercise test.

#### Lactate Diagnostic

For blood lactate diagnostics, 20 μL of capillary blood was collected from the earlobe five minutes before, immediately and three minutes after CPET termination. Blood lactate concentration was analyzed immediately post-exercise using the polarographic method with the EKF-BIOSEN-S-Line system (EKF-diagnostic GmbH, Magdeburg, Germany). Peak blood lactate was defined as the higher individual value obtained either immediately after exercise termination or after the 3-min recovery period. The blood lactate accumulation rate was calculated according to the method described by Cohen et al. 1947 [43], quantifying the net increase in blood lactate concentration relative to exercise duration.

### Statistical Analysis

Raw data were processed and analyzed using R (version 4.4.3, R Foundation for Statistical Computing, Boston, USA) within RStudio IDE (version 2025.05.1 + 513, RStudio, Inc., Boston, USA).

Continuous variables are reported as mean ± standard deviation, and categorical variables as absolute counts and percentages. Normality of continuous variables and homogeneity of variances were assessed using the Shapiro–Wilk test and Levene’s test, respectively. Depending on normality and homogeneity, we performed group comparisons using parametric (unpaired T-Test, Welch’s T-Test) or non-parametric methods (Mann–Whitney U test, Wilcoxon rank sum exact test). To assess associations between variables Spearman's rank correlation coefficients were computed for non-normally distributed variables, while Pearson's correlation coefficients were used for those with normal distributions. Relationships between ordinal variables were analyzed with the chi-squared test or, if expected cell counts were small, with Fisher’s exact test. All statistical tests were conducted with the *stats* package (v4.4.3). Additionally, repeated-measurement correlation analysis was conducted to account for variability in intercepts across individuals when multiple measurements from the same individuals were included (*rmcorr* package, v0.7.0). The significance level was set at α = 0.05, with all tests conducted two-sided.

## Results

### General Characteristics

The sample of 92 patients showed a high proportion of females (88%) and a high prevalence of obesity: 40 patients (43.5%) had a body mass index ≥ 30 kg/m^2^. On average, resting hemodynamic and pulmonary function values were within the normal range. However, 12 patients showed an elevated resting HR (> 80 bpm), and 16 patients had elevated blood pressure (systolic ≥ 140 mmHg and/or diastolic ≥ 90 mmHg). Two patients had a FVC below 40% predicted, and four had a FEV_1_ below 50% predicted. The mean PEF was slightly reduced to 81.5 ± 24.8% of the predicted value, with 40 patients having a PEF below 80% of the predicted value. No significant differences were observed between patients with and without ME/CFS (Table 2).

**Table 2:** Anthropometric characteristics, resting cardiovascular and pulmonary function data

	Total	PCC without ME/CFS	PCC with ME/CFS	
	N = 92^*1*^	N = 47 (51%)^*1*^	N = 45 (49%)^*1*^	p-value^*2*^
Sex				
Female	81 (88%)	41 (87%)	40 (89%)	0.8
Age [y]	50.7 ± 10.1	50.9 ± 9.9	50.6 ± 10.3	0.8
Body height [cm]	167.2 ± 8.2	167.4 ± 7.9	167.0 ± 8.6	0.6
Body mass [kg]	84.4 ± 23.6	84.8 ± 23.4	84.0 ± 24.2	0.7
BMI [kg/m^2^]	29.7 ± 7.8	29.9 ± 8.2	29.5 ± 7.4	> 0.9
Resting HR [1/min]	70.3 ± 10.6	70.1 ± 9.8	70.6 ± 11.5	> 0.9
BP_sys_ [mmHg]	125.5 ± 9.9	125.7 ± 11.1	125.3 ± 8.7	0.9
BP_dia_ [mmHg]	80.3 ± 6.1	81.0 ± 7.3	79.5 ± 4.4	0.3
FVC [L]	3.5 ± 1.0	3.5 ± 1.0	3.4 ± 1.0	0.7
FVC [%pred]	105.3 ± 22.8	105.6 ± 24.2	104.9 ± 21.5	> 0.9
FEV_1_ [L]	2.7 ± 0.8	2.8 ± 0.8	2.6 ± 0.7	0.4
FEV_1_ [%pred]	98.1 ± 21.7	99.9 ± 22.9	96.2 ± 20.5	0.4
PEF [L/s]	5.5 ± 1.8	5.7 ± 1.8	5.2 ± 1.7	0.2
PEF [%pred]	81.5 ± 24.8	84.5 ± 25.7	78.3 ± 23.7	0.2

^1^Data are presented as mean ± standard deviation or absolute numbers (N) and relative frequencies (%)

^2^Between-group comparisons were performed using Pearson’s chi-squared test for categorical variables and the Wilcoxon rank-sum test for continuous variables

Statistical significance was set at p < 0.05

*PCC* post-COVID-19 condition, *ME/CFS* myalgic encephalomyelitis/chronic fatigue syndrome, BMI body mass index, *HR* heart rate, *BP*_*sys*_systolic blood pressure, *BP*_*dia*_ diastolic blood pressure, *FVC* forced vital capacity, %pred percent of predicted value based on age, sex, and body mass, *FEV₁* forced expiratory volume in 1 s, *PEF* peak expiratory flow

### Clinical Profile

Acute SARS-CoV-2 infection occurred between 1 January 2020 and 27 March 2023. Most patients reported moderate (49%) or severe (40%) acute symptoms. Nine patients required hospitalization due to acute COVID-19, with a mean duration of 10.3 ± 7.4 days. None underwent extracorporeal membrane oxygenation.

The mean PCC symptom duration was 23.9 ± 7.5 months, with patients reporting an average of 6 ± 2 distinct PCC-related symptoms. The most frequently reported symptoms were fatigue (86%), brain fog (76%), dyspnea (55%), myalgia (54%), and headaches (38%). Based on the CCC, 45/92 patients (49%) met diagnostic criteria for ME/CFS. A high prevalence of PEM was observed: 71/92 patients (79%) screened positive on the DSQ-PEM, with higher prevalence in PCC patients with ME/CFS compared to those without (93% vs. 64%, χ^2^ = 9.6, p = 0.002).

Most patients had at least one pre-existing disease (84/92; 91%) with an average of 3 ± 2 different diseases. Most of the diseases were metabolic (58%), cardiovascular (36%) or pulmonary (21%). When comparing patients with and without ME/CFS, there were no significant differences in self-reported symptoms, with fatigue being the most prevalent symptom in both conditions without ME/CFS and with ME/CFS. There were no significant differences regarding the comorbidity classes between the two groups although a higher proportion of patients with ME/CFS (22.2%) demonstrated at least one severe comorbidity compared to those without (4.3%). A greater proportion of patients without ME/CFS (55.3%) had no or fewer than three mild comorbidities, in contrast to the proportion observed in patients diagnosed with ME/CFS (33.3%).

When combining the prevalence of severe comorbidities and three or more comorbidities, a trend towards a higher prevalence of multimorbidity in the ME/CFS group compared to PCC patients without ME/CFS was observed (p = 0.056). In detail, 30/45 patients (67%) with ME/CFS had either three or more comorbidities or at least one severe comorbidity compared to 21/47 patients (45%) without ME/CFS. A complete list of all comorbidities and their prevalence can be found in Supplementary File 4.

### Exercise Physiological Profile

In the following analysis, patients were stratified by ME/CFS status, multimorbidity and/or severe comorbidities to assess physiological limitations.

#### Cardiorespiratory Fitness

The overall group demonstrated marked impairments in physical capacity, with a VO_2peak_ of 14.2 ± 4.3 mL/min/kg and a reduced ratio of actual VO_2peak_ to individually predicted VO_2peak_ (%pred, adjusted for sex, age and body mass) of 66.9 ± 16.9%. From the entire patient group 75/92 patients (81.5%) demonstrated a VO_2peak_ below 80% of the predicted value and 32/92 patients (34.8%) exhibited a value below 60% of the predicted value. Mean maximum HR was 133 ± 23 bpm, which does not indicate cardiac exertion. The mean respiratory exchange ratio (RER: 1.06 ± 0.1) indicates substantial anaerobic strain, with 49/92 patients (53%) having a RER of ≥ 1.05, 21/92 patients (23%) having a RER of 1.0–1.04 and a subset of 22/92 patients (24%) did not achieve a RER ≥ 1.0.

PCC patients with ME/CFS demonstrated significantly lower VO_2peak_ (13.0 ± 3.1 vs. 15.4 ± 4.9 mL/min/kg, p = 0.012) and reduced predicted VO_2peak_ (61.9 ± 16.7% vs. 71.6 ± 16.0%, p = 0.003) compared to patients without ME/CFS. At submaximal intensities VO_2_ at VT1 (8.5 ± 2.1 vs. 9.4 ± 2.2, p = 0.052) and VT2 (11.4 ± 2.8 vs. 13.2 ± 3.3, p = 0.009) was reduced in PCC patients with ME/CFS. Ratios of VO_2_ at VT1 and VT2 to their predicted VO_2peak_ were also reduced in patients with ME/CFS (VT1: 40.9 ± 8.1 vs. 44.5 ± 8.8%, p = 0.043; VT2: 56.1 ± 12.6 vs. 60.8 ± 11.7%, p = 0.075).

When comparing the cohort of those with multimorbidity/severe comorbidity to those with less than three mild to moderate and no severe comorbidities, differences in VO_2_ were not significant after adjustment for sex, age, and body mass (Table 3).

**Table 3:** Results of cardiorespiratory exercise testing for all patients, divided by ME/CFS and multimorbidity status

	Total	ME/CFS			Severe comorbidity/multimorbidity (≥ 3)		
	N = 92^*1*^	no, N = 47 (51%)^*1*^	yes, N = 45 (49%)^*1*^	p-value	no, N = 41 (45%)^*1*^	yes, N = 51 (55%)^*1*^	p-value
Cardiorespiratory fitness							
Time_peak_ [s]	767.1 ± 277.7	855.3 ± 294.4	675.0 ± 227.8	** < 0.001**^*2*^	813.5 ± 339.1	729.8 ± 212.6	0.2^*2*^
HR_peak_ [bpm]	132.6 ± 22.9	136.9 ± 24.2	128.2 ± 20.7	0.067^*3*^	137.8 ± 25.3	128.5 ± 19.9	0.051^*3*^
VO_2VT1_ [mL/min/kg]	9.0 ± 2.2	9.4 ± 2.2	8.5 ± 2.1	0.052^*4*^	9.8 ± 2.4	8.3 ± 1.8	**0.001**^*2*^
VO_2VT1_ [%pred]	42.8 ± 8.6	44.5 ± 8.8	40.9 ± 8.1	**0.043**^*3*^	41.8 ± 10.0	43.6 ± 7.4	0.3^*3*^
VO_2VT2_ [mL/min/kg]	12.4 ± 3.2	13.2 ± 3.3	11.4 ± 2.8	**0.009**^*4*^	13.7 ± 3.7	11.5 ± 2.5	**0.004**^*4*^
VO_2VT2_ [%pred]	58.6 ± 12.3	60.8 ± 11.7	56.1 ± 12.6	0.075^*4*^	57.0 ± 13.9	59.7 ± 11.0	0.2^*4*^
VO_2peak_ [mL/min/kg]	14.2 ± 4.3	15.4 ± 4.9	13.0 ± 3.1	**0.012**^*4*^	15.6 ± 5.1	13.2 ± 3.3	**0.011**^*2*^
VO_2peak_ [%pred]	66.9 ± 16.9	71.6 ± 16.0	61.9 ± 16.7	**0.003**^*4*^	65.0 ± 19.5	68.3 ± 14.7	0.2^*2*^
Rate of perceived exertion							
RPE_VT1_	12.9 ± 3.0	12.3 ± 3.1	13.6 ± 2.9	**0.033**^*3*^	12.8 ± 3.3	13.0 ± 2.8	0.7^*3*^
RPE_VT2_	16.0 ± 2.4	15.4 ± 2.5	16.7 ± 2.1	**0.025**^*2*^	15.8 ± 2.8	16.2 ± 2.0	0.8^*2*^
RPE_peak_	18.4 ± 1.2	18.3 ± 1.5	18.6 ± 0.9	0.7^*2*^	18.5 ± 1.5	18.4 ± 1.1	0.3^*2*^
Power output							
Power_VT1_ [W]	41.1 ± 8.3	43.1 ± 8.0	39.0 ± 8.2	**0.004**^*2*^	42.5 ± 9.8	40.0 ± 6.8	0.4^*2*^
Power_VT2_ [W]	64.5 ± 18.5	69.8 ± 20.0	58.7 ± 15.0	**0.003**^*2*^	71.4 ± 22.8	59.7 ± 13.2	**0.028**^*2*^
Power_peak_ [W]	78.2 ± 27.3	86.3 ± 31.1	69.7 ± 19.7	**0.001**^*2*^	84.3 ± 35.0	73.2 ± 18.1	0.13^*2*^
Power_peak_ [W/kg]	1.0 ± 0.4	1.1 ± 0.5	0.9 ± 0.3	**0.014**^*2*^	1.1 ± 0.5	0.9 ± 0.3	**0.008**^*2*^
Power_peak_ [%pred]	57.2 ± 19.4	62.7 ± 21.2	51.5 ± 15.7	**0.002**^*2*^	59.1 ± 23.4	55.7 ± 15.6	0.5^*2*^
Oxygen uptake efficiency							
OUES	579.2 ± 154.4	609.3 ± 144.3	547.8 ± 159.8	0.056^*3*^	589.6 ± 162.1	570.9 ± 148.9	0.6^*3*^
O_2_ pulse_VT1_ [mL/bpm]	7.5 ± 1.7	7.8 ± 1.7	7.1 ± 1.6	**0.043**^*4*^	7.7 ± 2.0	7.2 ± 1.4	0.2^*5*^
O_2_ pulse_VT2_ [mL/bpm]	8.4 ± 1.8	8.7 ± 1.8	8.2 ± 1.8	0.2^*4*^	8.6 ± 2.1	8.3 ± 1.5	0.7^*4*^
O_2_ pulse_peak_ [mL/bpm]	8.1 ± 2.0	8.5 ± 1.9	7.7 ± 2.0	**0.047**^*3*^	8.1 ± 2.2	8.1 ± 1.8	0.7^*2*^
Metabolic response							
RER_peak_	1.1 ± 0.1	1.1 ± 0.1	1.0 ± 0.1	0.4^*3*^	1.1 ± 0.1	1.1 ± 0.1	0.9^*2*^
Lactate_rest_ [mmol/L]	1.0 ± 0.3	0.9 ± 0.3	1.0 ± 0.3	0.8^*2*^	1.0 ± 0.3	0.9 ± 0.3	0.8^*2*^
Lactate_peak_ [mmol/L]^6^	3.2 ± 2.0	3.7 ± 2.3	2.7 ± 1.4	**0.022**^*2*^	3.5 ± 2.5	3.0 ± 1.4	> 0.9^*2*^
Lactate rate_peak_ [mmol/L/s]^7^	0.0041 ± 0.0018	0.0043 ± 0.0020	0.0040 ± 0.0015	0.7^*2*^	0.0042 ± 0.0021	0.0040 ± 0.0014	0.9^*2*^
Ventilatory response							
VE/VCO_2_ Slope	37.7 ± 9.0	37.8 ± 5.7	37.6 ± 11.5	0.5^*4*^	36.6 ± 8.1	38.6 ± 9.6	0.8^*2*^
PETCO_2rest_ [mmHg]	30.9 ± 3.3	31.2 ± 2.4	30.5 ± 4.1	> 0.9^*4*^	30.4 ± 3.5	31.3 ± 3.1	0.3^*2*^
PETCO_2VT2_ [mmHg]	34.4 ± 3.7	34.6 ± 3.1	34.1 ± 4.3	0.6^*3*^	34.7 ± 2.9	34.1 ± 4.2	0.5^*5*^
VE/VO_2peak_	38.2 ± 8.4	38.2 ± 6.3	38.1 ± 10.1	0.5^*4*^	38.3 ± 6.4	38.1 ± 9.7	0.6^*2*^
VE/VCO_2peak_	36.1 ± 5.8	35.9 ± 4.3	36.3 ± 7.1	0.9^*4*^	36.2 ± 4.4	35.9 ± 6.8	0.6^*2*^

^1^Data are presented as mean ± standard deviation. Between-group comparisons were conducted using ^2^Wilcoxon rank-sum test, ^3^Two-sample t-test, ^4^Wilcoxon rank-sum exact test or ^5^Welch two-sample t-test, as appropriate. ^6^Lactate_peak_ represents the higher value measured at exercise termination or 3-min recovery. ^7^Lactate rate_peak_: peak lactate concentration divided by total exercise time

Statistical significance was set at p < 0.05; significant differences are shown in bold

*ME/CFS* myalgic encephalomyelitis/chronic fatigue syndrome, *VO*_*₂peak*_ peak oxygen uptake, *VO₂* oxygen uptake, *PPO* peak power output, *Time*_*peak*_ total exercise time, *HR*_*peak*_ peak heart rate, VT1 and *VT2* first and second ventilatory threshold, *%pred* percent of predicted value based on age, sex, and body mass, *RPE* rate of perceived exertion, *OUES* oxygen uptake efficiency slope, *O₂* pulse oxygen pulse,, *RER*_*peak*_ peak respiratory exchange ratio, *VE/VCO₂* ventilatory equivalent for carbon dioxide, *PETCO₂* end-tidal partial pressure of carbon dioxide

#### Rate of Perceived Exertion

Mean RPE at the point of exercise termination was 18.4 ± 1.2, but 7/92 patients (7.6%) did not reach a RPE of 17. No differences at termination between PCC patients with and without ME/CFS were observed. At submaximal intensities, patients with ME/CFS reported higher RPE at VT1 (13.6 ± 2.9 vs. 12.3 ± 3.1, p = 0.033) and VT2 (16.7 ± 2.1 vs. 15.4 ± 2.5, p = 0.025) than those without ME/CFS. Repeated-measures correlation revealed strong associations between RPE and VO₂ (r = 0.82, p < 0.001), HR (r = 0.87, p < 0.001) and PO (r = 0.80, p < 0.001), indicating close alignment between exertion perception and physiological responses in this cohort.

#### Power Output

Absolute and relative mean PPO at exhaustion was markedly reduced, with PCC patients with ME/CFS having significantly lower absolute PPO (69.7 ± 19.7 vs. 86.3 ± 31.1 W, p = 0.001) and relative PPO (0.9 ± 0.3 vs. 1.1 ± 0.5 W/kg, p = 0.014) than those without ME/CFS. Only relative PPO differed significantly between patients with multimorbidity and those without multimorbidity (0.9 ± 0.3 vs. 1.1 ± 0.5 W/kg, p = 0.008). The PPO [%pred] was also significantly lower in PCC patients with ME/CFS compared to those without ME/CFS (51.5 ± 15.7 vs. 62.7 ± 21.2%, p = 0.002) and was not significantly different between patients with multimorbidity and those without multimorbidity (55.7 ± 15.6 vs. 59.1 ± 23.4%, p = 0.5). At submaximal intensities, PCC patients with ME/CFS demonstrated significantly lower absolute PO compared to patients without ME/CFS (VT1: p = 0.004, VT2: p = 0.003).

#### Oxygen Uptake Efficiency

Oxygen uptake efficiency during exercise was impaired, as indicated by an OUES of 579.2 ± 154.4 mL/min and by a reduced O_2_ pulse at VT1 (7.5 ± 1.7 mL/bpm), VT2 (8.4 ± 1.8 mL/bpm), and exhaustion (8.1 ± 2 mL/bpm).

PCC patients with ME/CFS demonstrated a significantly lower O_2_ pulse at VT1 (7.1 ± 1.6 vs. 7.8 ± 1.7 mL/bpm, p = 0.043) and exhaustion (7.7 ± 2.0 vs.8.5 ± 1.9 mL/bpm, p = 0.047) in comparison to patients without ME/CFS. Furthermore, there was a trend towards a lower OUES in PCC patients with ME/CFS compared to those without ME/CFS (547.8 ± 159.8 vs. 609.3 ± 144.3, p = 0.056).

#### Metabolic Response

Mean resting lactate concentration was 1 ± 0.3 mmol/L, with no significant differences by ME/CFS or comorbidity status. Half of the PCC patients 46/92 (50%) did not reach lactate levels above 3 mmol/L at exhaustion, with an even higher percentage in patients with ME/CFS (60%) compared to those without ME/CFS (40.4%).

PCC patients without ME/CFS had significantly higher concentrations of lactate at exhaustion (3.9 ± 2.4 vs 2.9 ± 1.5, p = 0.043). However, when normalized to exercise duration (mmol/L/s), no significant differences remained (p = 0.8).

#### Ventilatory Response

The large majority of patients exhibited indications of hyperventilation at rest and during exercise, with 85/92 patients (92.4%) exceeding a VE/VCO_2_ slope of 30, and 87/92 patients (94.6%) having a PETCO_2_ below 36 mmHg at rest. Furthermore, 31/92 patients (33.7%) demonstrated a maximal exercise increase in PETCO_2_ below 3 mmHg. There was no significant difference in the ventilatory equivalents between PCC patients with or without ME/CFS. Only four patients exhibited a normal ventilatory response at rest and during exercise.

### Diagnostic Utility of CPET for Physical Work Ability

This section provides a detailed analysis of the physical work ability in PCC patients based on CPET-derived well-established physiological thresholds.

#### Functional Assessment of Physical Capacity

**Fig. 3:**
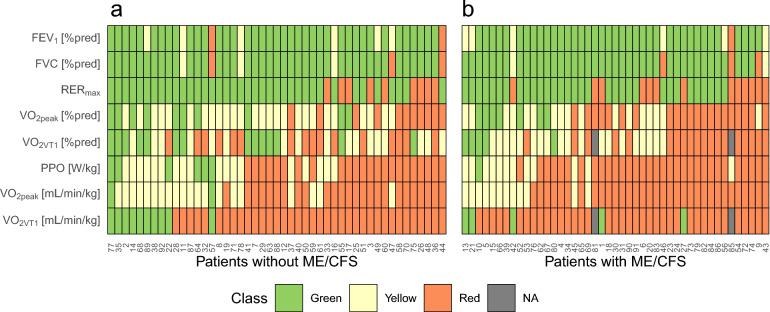
Classification of physical work ability across all PCC patients stratified by ME/CFS diagnosis. **a** Patients without ME/CFS diagnosis. **b** Patients with ME/CFS diagnosis. Classifications are based on resting pulmonary function test and spiroergometric parameters. Each tile represents the classification of an individual participant’s result for a given physiological variable. Color coding indicates performance thresholds: FEV₁ [%pred]: Green ≥ 80%, Yellow = 79–41%, Red ≤ 40%. FVC [%pred]: Green ≥ 80%, Yellow = 79–50%, Red < 50%. VO₂_peak_ [%pred]: Green > 80%, Yellow = 60–80%, Red < 60%. VO₂_peak_ [mL/min/kg]: Green ≥ 25, Yellow = 15–24.9, Red < 15. PPO [W/kg body mass]: Green > 1.5, Yellow = 1.0–1.5, Red < 1.0. VO₂_VT1_ [%pred]: Green > 50%, Yellow = 40–50%, Red < 40%. VO₂_VT1_ ≥ 11 [mL/min/kg]: Green ≥ 11, and Red < 11. Grey = data not available due to terminating CPET before reaching VT1. *PCC* post-COVID-19 condition; *ME/CFS* myalgic encephalomyelitis/chronic fatigue syndrome; *FEV₁* forced expiratory volume in 1 s, *%pred* percent of predicted value based on age, sex, and body mass, *FVC* forced vital capacity, *VO₂*_*peak*_ peak oxygen uptake, *PPO* peak power output, *VT1* first ventilatory threshold

Analyzing resting and exercise data obtained during CPET, we identified several physiological parameters indicating limitations in physical work ability (Fig. 3).

Pulmonary function tests at rest revealed that 9/92 patients (9.8%) demonstrated considerably limited results with FVC < 50%pred and/or FEV_1_ ≤ 40%pred. Twenty-two patients (23.9%) had to terminate the CPET before reaching a RER of ≥ 1.0, a commonly applied threshold for sufficient effort in patient cohorts [40]. Of those patients, 7 had elevated VE/VO_2_ (≥ 35) at exercise termination, suggestive of ventilatory strain and adequate patient effort. Two patients with ME/CFS had to terminate the CPET even before reaching their VT1. Both patients had a Bell-Score of 30 points or less. Subsequent analyses are presented for the entire cohort and for the subgroup achieving RER ≥ 1.0 (RER +). In contrast to resting pulmonary function, CPET showed that 58/92 patients (63%) had a VO_2peak_ below 15 mL/min/kg (52.9% in RER +), which is a common cut-off value for the ability to work. Further, 35/92 patients (38%) had a VO_2_ at VT1 (VO_2VT1_) below 40% of the predicted value (38.6% in RER +) and 55/92 patients (59.8%) had a load below 1 W/kg (51.4% RER +). Fifty-seven patients (81.5%) did not reach a VO_2_ of 11 mL/min/kg at their VT1 (80% in RER +). Overall, 82 patients (89.1%) had at least one red category in one of the analyzed variables, which means that the lowest threshold value was not achieved (74.3% in RER +). The percentage of patients with ME/CFS who had at least one red category was 95.6% (Fig. 3b), compared to 83% of patients without ME/CFS (Fig. 3a). Only one patient without ME/CFS exhibited normal values across all parameters.

#### Association Between CPET and Bell Disability Scale

The mean Bell-Score reported by 88 patients was 35.5 ± 13.3, indicating moderate-to-severe functional impairment. Almost half of the patients (48.9%) had a Bell-Score of 30 or less. PCC patients with ME/CFS had a significantly lower Bell-Score than those without ME/CFS (31.6 ± 10.3 vs. 39.4 ± 14.9, p = 0.001).

To compare subjective work disability (Bell Disability Scale) with objectivly CPET-derived measures, patients were dichotomized by Bell-Score (≤ 30 vs. > 30) and by physiological thresholds for VO₂ and PPO (Table 4). Chi-squared analyses revealed significant associations of Bell-Scores with both VO₂_peak_ and PPO in the total cohort (p = 0.017 and 0.019, respectively) and in the RER + subgroup (both p = 0.003, see Supplementary File 5).

**Table 4:** Association between bell-score with submaximal and maximal CPET parameters and CPET-derived work ability status

	Bell-score		
	> 30^1^	< = 30^1^	P-value^2^
VO_2VT1_ [mL/min/kg]			
≥ 11	10 (23.3%)	4 (8.9%)	
< 11	33 (76.7%)	39 (86.7%)	0.144
VO_2VT1_ [%pred]			
≥ 45	20 (46.5%)	12 (26.7%)	
< 45	23 (53.5%)	31 (68.9%)	0.118
VO_2peak_ [mL/min/kg]			
≥ 15	21 (48.8%)	10 (22.2%)	
< 15	22 (51.2%)	35 (77.8%)	**0.017**
VO_2peak_ [%pred]			
≥ 60	32 (74.4%)	24 (53.3%)	
< 60	11 (25.6%)	21 (46.7%)	0.067
PPO [W/kg]			
≥ 1.0	23 (53.5%)	12 (26.7%)	
< 1.0	20 (46.5%)	33 (73.3%)	**0.019**
Work ability (CPET)			
Yes	21 (48.8%)	8 (17.8%)	
No	22 (51.2%)	37 (82.2%)	**0.004**

^1^Data are presented as absolute numbers (N) and relative frequencies (%).

^2^Between-group comparisons were conducted using Chi-squared analyses.

Statistical significance was set at p < 0.05; significant differences are shown in bold

*VO₂*_*peak*_ peak oxygen uptake, *PPO* peak power output, *VT1* first ventilatory threshold, *%pred* percent of predicted value based on age, sex, and body mass, *W/kg* watts per kilogram body mass, *Work ability (CPET)* was classified as “no” for participants with VO₂_peak_ < 15 mL/min/kg and/or PPO < 1.0 W/kg body mass, *CPET* cardiopulmonary exercise testing

The association with the Bell-Score increased further when VO₂_peak_ < 15 mL/min/kg or PPO < 1.0 W/kg were summarized as combined thresholds. In this case, significant associations were observed for the total cohort (p = 0.004) and the RER + subgroup (p < 0.001). Using both VO₂_peak_ and PPO as criteria to detect work disability, 82.2% of the patients with a Bell-Score ≤ 30 could be identified (79.4% in the RER +).

Spearman’s rank correlation revealed significant associations between the Bell-Score and VO_2peak_ (ρ = 0.30, p = 0.005) and PPO (ρ = 0.31, p = 0.003) in the entire cohort, with even stronger associations in the RER + group (VO_2peak_: ρ = 0.41, p < 0.001 & PPO: ρ = 0.45, p < 0.001). No significant correlation was observed between Bell-Score and VO_2VT1_ (p = 0.22)_._ All correlations between the Bell-Score and CPET-derived parameters are provided in Supplementary File 6.

#### Occupational Status in Relation to Functional Capacity

**Fig. 4:**
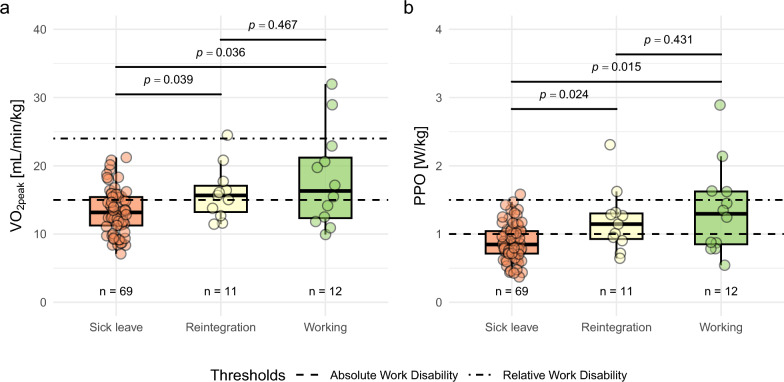
Differences in self-reported work status and exercise capacity. **a** Differences in VO_2peak_ and **b** differences in PPO across groups of patients reporting sick leave, reintegration, or working. Between-group comparisons were performed using the Kruskal–Wallis test, followed by post hoc pairwise comparisons with Bonferroni–Holm–adjusted p-values. Statistical significance was set at p < 0.05. *VO₂peak* peak oxygen uptake, *PPO* peak power output

To investigate the relationship between occupational status and objective markers of physical capacity, we analyzed VO_₂peak_ [mL/min/kg] and PPO [W/kg body mass] across three groups: 69/92 patients (75%) on medical sick leave, 11/92 patients (12%) in work reintegration, and 12/92 patients (13%) currently working (Fig. 4).

VO_2peak_ and PPO were the lowest among patients on medical sick leave (13.3 ± 3.3 mL/min/kg and 0.9 ± 0.3 W/kg), compared to patients in reintegration (16.0 ± 3.9 mL/min/kg, p = 0.039 and 1.2 ± 0.5 W/kg, p = 0.024) and those currently working (18.0 ± 7.1 mL/min/kg, p = 0.036 and 1.2 ± 0.5 W/kg, p = 0.015). These findings remained consistent when analyzed using either the complete dataset or the RER + subgroup. No significant difference was found between the reintegration and working groups. Additionally, patients on sick leave had mean VO₂_peak_ and mean PPO values below the absolute physical work disability thresholds of 15 mL/min/kg and 1.0 W/kg, respectively. By contrast, patients undergoing reintegration and those in employment exceeded these thresholds but remained below the levels considered necessary for full work ability (VO₂_peak_ ≥ 25.0 mL/min/kg; PPO ≥ 1.5 W/kg).

## Discussion

### Principal Findings

We present a comprehensive characterization of severely and long-term affected PCC patients, integrating PROMs, clinical assessments, and CPET to provide a robust framework for objectively evaluating work ability. To our knowledge, this is the first study to demonstrate pronounced functional impairments with significant implications for work ability in this patient population.

PCC patients with ME/CFS exhibited significantly lower VO₂_peak_ and PPO as well as markedly diminished O₂ pulse values adjusted for sex, age, and body mass. These impairments cannot be explained by the presence of comorbidities or deconditioning alone and support the hypothesis that peripheral functional limitations, likely driven by impaired systemic oxygen extraction rather than central structural organ abnormalities, represent a predominant physiological mechanism.

A substantial proportion of participants met the criteria for work disability, either objectively through CPET or subjectively via the Bell Disability Scale. Notably, there was a high concordance between subjective and objective indicators of work ability. VO₂_peak_ and PPO also varied significantly by occupational status, with the lowest values observed in patients on medical sick leave.

Taken together, our findings underscore the utility of CPET as a valid, non-invasive tool for quantifying physical work ability and characterizing physiological limitations relevant to occupational functioning in PCC and ME/CFS. These insights have direct implications for clinical assessment and return-to-work planning.

### Clinical Profile

Our cohort had a mean age of 50.7 years and was predominantly female (88%), with an average body mass index of 29.7 kg/m^2^ and almost three comorbidities per patient. These findings align with prior research identifying middle age, female sex, obesity, and comorbidities as key risk factors for PCC development [44]. This finding highlights multimorbidity as an additional risk factor for particularly long-standing and severe PCC [44, 45]. The multimorbidity rate (42%) exceeded the 25.3% reported in age-matched populations [46, 47], and surpassed individual disease category estimates [48]. Interestingly, only the presence of severe comorbidities differed significantly between PCC patients with and without ME/CFS (22.2% vs. 4.3%), while the total number of comorbidities was almost identical. The mean PCC related symptom duration was approximately 24 months, and the most commonly reported symptoms included fatigue, brain fog, hyperventilation, myalgias and headaches. Equally noteworthy, 49% of our PCC cohort met the CCC for ME/CFS, a proportion markedly higher than the 9.8%–15.4% reported in international studies [9–11]. This discrepancy likely reflects a recruitment bias, as our study focused on long-term and severely affected PCC patients with a probable occupational disease status due to SARS-CoV-2 infection. Additionally, a high prevalence of PEM was observed in both sub-cohorts with ME/CFS and without ME/CFS (93% vs. 64%). This underscores the unique nature of this work, which to our knowledge is the first to fully describe disease burden and objectively measured limitations of physical work ability using CPET in long-term and severely affected PCC patients.

### Exercise Physiological Profile

Patients with PCC exhibited markedly reduced physical capacity, reflected by a mean VO₂_peak_ of 14.2 mL/min/kg (67% of predicted), and a mean PPO of 78.2 W (57% of predicted). When stratified by ME/CFS status, patients meeting diagnostic criteria for ME/CFS showed significantly lower performance than patients without ME/CFS: VO₂_peak_ (13.0 vs. 15.4 mL/min/kg), and PPO (69.7 vs. 86.3 W), indicating more pronounced impairment in this subgroup. Additionally, the ME/CFS group reported lower Bell-Score (31.6 vs. 39.4), further underscoring their functional impairment. However, no significant differences in physical capacity were found between patients with and without multimorbidity or severe comorbidities when analyzing observed-to-predicted ratios. This may be partly explained by the higher prevalence of obesity in the multimorbidity group (55% vs. 32%), suggesting that normalization to body mass attenuates potential differences. It is important to note that, although 92.4% of patients reported a subjective rate of perceived exertion of ≥ 17, 50% of patients did not surpass the lactate threshold of 3 mmol/L, and 23.9% failed to reach an RER of 1.0 suggesting that CPET termination was due to symptom exacerbation, rather than metabolic exhaustion. This pattern reflects acute exercise intolerance that is not strictly workload-dependent. Additionally, patients with ME/CFS had significantly higher RPE at VT1 and VT2, despite lower power output at both ventilatory thresholds compared to patients without ME/CFS. These findings indicate an earlier onset of symptom-limited exhaustion in the most severely affected patients and underscore the need to assess subjective burden alongside physiological markers.

In addition to the markedly reduced physical capacity observed across the PCC cohort, patients exhibited a pronounced abnormal ventilatory response. Abnormalities in ventilatory efficiency were pervasive. The mean VE/VCO₂ slope was elevated at 37.7 ± 9.0, with 92% of patients exceeding the pathological threshold of 30. In addition, nearly all patients (95%) had resting PETCO₂ values below 36 mmHg, averaging 30.9 ± 3.3 mmHg, and one-third (34%) exhibited a blunted PETCO₂ response to exercise (< 3 mmHg increase). Ventilatory equivalents at peak load were also elevated (VE/VO₂: 38.2 ± 8.4; VE/VCO₂: 36.1 ± 5.8), reinforcing the presence of dysfunctional ventilatory regulation under stress. These findings are consistent with chronic hyperventilation or ventilatory control disturbances, which may contribute to dyspnea and exercise intolerance [49, 50]. Interestingly, there was no significant difference in ventilatory abnormalities between patients with and without ME/CFS or between those with and without multimorbidity or severe comorbidities. In contrast to our findings, Mancini et al. reported a lower, yet still, relevant prevalence of hyperventilation (32%) and dysfunctional breathing (42%) in ME/CFS, which may partly reflect the higher physical capacity, younger age, and lower body mass index of their cohort compared with the present PCC sample [51]. Both hyperventilation and dysfunctional breathing have been linked to autonomic dysregulation, characterized by increased sympathetic and reduced parasympathetic activity [52, 53]. Although direct evidence remains limited, it is plausible that severe or recurrent respiratory infections, as reflected by nine hospitalizations in our cohort, may contribute to the development or persistence of ventilatory dysfunction and hyperventilation in PCC [54]; however, such mechanisms are unlikely to apply uniformly across all post-infectious fatigue syndromes. Taken together, these findings highlight dysfunctional ventilatory control as a clinically relevant and potentially modifiable contributor to exercise intolerance in PCC, supporting the integration of targeted breathing therapy focusing on CO₂ tolerance and ventilatory efficiency as a complementary component of exercise-based rehabilitation [54, 55].

Importantly, objective signs of cardiopulmonary impairment at rest were rare in this cohort. Only 10% of patients demonstrated pulmonary limitation on body plethysmography (FVC < 50% in six; FEV₁ < 40% in three), and no pathological findings were observed on resting ECGs. This argues against structural cardiopulmonary disease as the primary cause of exercise intolerance.

Instead, several parameters suggest impaired systemic oxygen extraction as key limiting mechanisms. Physiologically, O₂ pulse reflects the product of stroke volume and the arterio-venous oxygen difference, thereby serving as a non-invasive surrogate of systemic oxygen extraction, particularly in the absence of central cardiac pathology [39, 40]. In the present study, O₂ pulse was derived non-invasively via spiroergometry and was found to be significantly reduced at all levels of exertion. This impairment was more pronounced in patients fulfilling ME/CFS criteria. Compared to non-ME/CFS patients, they exhibited significantly lower O₂ pulse at VT1 (7.1 ± 1.5 vs. 7.8 ± 1.7 mL/bpm, p = 0.043) and at peak exercise (7.7 ± 2.0 vs. 8.5 ± 1.9 mL/bpm, p = 0.047). These values are well below normative values and point to reduced circulatory or muscular oxygen utilization [20, 22], reinforcing the hypothesis of peripheral dysfunction. Further evidence of impaired oxygen uptake efficiency was provided by mean OUES, which was 579.2 ± 154.4 mL/min. This was markedly lower than the expected values for healthy individuals and indicated inefficient ventilatory response to increasing metabolic demand [56]. Together, these findings suggest that functional limitations in this cohort are primarily driven by suboptimal oxygen uptake efficiency rather than central cardiac or pulmonary pathology. This aligns with prior studies: Singh et al. (2023) identified systemic inflammation and endothelial injury as drivers of impaired hemodynamics during exercise [57]; Baratto et al. (2021) reported post-COVID-19 vascular dysfunction limiting oxygen delivery [58]; and Schäfer et al. (2023) observed reduced tissue oxygenation linked to microcirculatory dysfunction [59]. Notably, Singh et al. (2022) and Kahn et al. (2024), using invasive CPET, also identified impaired systemic oxygen extraction as the primary mechanism of exertional intolerance in PCC [24, 60].

### Diagnostic Utility of CPET for Physical Work Ability

We provide the first in-depth assessment of CPET-derived work ability in a cohort of patients with long-standing, severe PCC and ME/CFS. In our sample, 63% of patients exhibited VO₂_peak_ values below 15 mL/min/kg, and 60% had a PPO below 1 W/kg—both thresholds associated with absolute physical work disability [21, 23]. Additionally, 40% of the patients had VO₂_VT1_ values below 40% of their estimated VO₂_peak_, and 81.5% of the patients fell below 11 mL/min/kg— submaximal thresholds linked also to work disability [19, 21]. These results indicate that submaximal CPET can still yield meaningful diagnostic insights into work ability.

Even patients within relative work ability (VO₂_peak_ between 15 and 25 mL/min/kg, PPO between 1 and 1.5 W/kg, or VO₂_VT1_ between 40 and 50%) are likely to experience substantial limitations [22, 23]. Routine tasks such as walking or tidying a bed can demand over 40% of VO₂_peak_, thereby exceeding sustainable physiological reserves in this group [61].

Most patients in the present cohort had previously been engaged in moderately demanding occupations within the fields of healthcare, social services, or education. However, it is improbable that these patients can now sustain full-time employment, even in reduced roles. In contrast, a large population-based study by Peter RS et al. (2025) found that PCC patients without ME/CFS reached higher VO₂_peak_ values (~ 27.9 mL/min/kg) two years post-infection [62], indicating preserved work capacity in less severely affected populations. Nevertheless, even these individuals showed reduced VO₂_peak_ compared to healthy controls [26, 62].

CPET showed high sensitivity in identifying work disability. The concordance between objective CPET thresholds and self-reported disability was strong: 82.5% of patients with a Bell-Score ≤ 30 were correctly classified by CPET. Spearman's correlation revealed low-to-moderate associations between the Bell-Score and both VO₂_peak_ and PPO. Furthermore, patients on medical sick leave showed significantly lower physical capacity than those undergoing reintegration or are still working. Their mean VO₂_peak_ and PPO values fell below thresholds for absolute work disability, while the other groups exceeded these values—though without meeting the criteria for full work ability (VO₂_peak_ ≥ 25 mL/min/kg or PPO ≥ 1.5 W/kg). There are numerous factors that motivate patients to continue working, including encompassing financial concerns, loan repayments, job loss, and the aspiration to contribute to society [63]. It is assumed that the working patients who remain below thresholds for absolute work ability are likely to encounter considerable constraints on their health-related quality of life [64].

However, CPET alone may be insufficient in some cases. While it captures key cardiorespiratory and peripheral impairments, additional factors including mental health, cognitive dysfunction, and symptom variability also limit work ability [7, 14]. Notably, 5 out of 92 patients (5.4%) showed near-normal VO₂_peak_ (≥ 80% of predicted) yet reported severe functional limitations (Bell-Score ≤ 30). Conversely, 2 patients (4.3%) failed to reach the VT1, limiting the assessment of even submaximal work ability. However, during prior clinical assessments, the two patients showed reduced pulmonary function and tachycardia, respectively. These discrepancies highlight the need for a multidisciplinary diagnostic approach that integrates CPET with clinical diagnostics and PROMs.

Despite its clinical and occupational value, CPET must be applied with caution in severely affected PCC and ME/CFS patients. As shown by Appelman et al. (2024), excessive exertion may trigger PEM and pathological muscle changes [65]. Leitner et al. (2024) also reported systemic oxygen extraction plateaus occur at ~ 4 metabolic equivalents (comparable to the mean VO₂_peak_ of 14.2 mL/min/kg observed in our cohort), beyond which anaerobic metabolism predominates—potentially marking a PEM threshold [28]. This metabolic shift leads to a significant lactate and H⁺ accumulation [66], increased reactive oxygen species [67], and energy depletion, contributing to symptom worsening and impaired recovery [68]. However, no acute adverse events occurred during or immediately after CPET.

Taken together, our findings support the use of CPET as a diagnostic procedure in patients with severe PCC and ME/CFS. When interpreted in conjunction with PROMs and standard clinical assessments, CPET enables individualized diagnostic and occupational decisions. In addition to detecting “invisible” functional impairments, CPET can objectively quantify reduced physical work ability.

## Limitations

When interpreting the results, certain factors need to be considered. All included participants were employed in social and healthcare sectors, leading to an overrepresentation of females. Additionally, our cohort included only individuals with severe and long-term PCC, which may not reflect the broader PCC population. The absence of healthy controls, matched for age and sex limits the generalizability of our findings. Another limitation is the missing evaluation of the bidirectional effects between PEM and CPET, since the study lacked a second consecutive CPET and systematic monitoring of PEM-related symptoms before and after CPET. Future studies should incorporate structured post-exercise monitoring to assess PEM risk and refine CPET protocols to enhance both safety and diagnostic value.

## Conclusion

This study is the first to demonstrate pronounced reductions in physical capacity among severely affected PCC patients, particularly in those meeting ME/CFS criteria.

Most patients (66.3%) had a VO_2peak_ < 15 mL/min/kg and/or a PPO < 1 W/kg, indicating absolute work disability, which was strongly associated with self-reported functional limitations and current occupational status.

In addition, VO₂ at submaximal load (VT1 < 11 mL/min/kg or < 40% of predicted VO₂) is capable to detect severely reduced physical work ability in patients unable to reach exhaustion due to symptom exacerbation.

When combined with PROMs and standard clinical assessments, CPET not only guides further organ-centered clinical diagnostics but also enables individualized therapy planning and informed return-to-work decisions.

## Supplementary Information

Supplementary Material 1.

## Data Availability

The datasets generated and/or analyzed during the current study are available from the corresponding author on reasonable request.

## Abbreviations

*PCC*: Post-COVID-19 condition
*ME/CFS*: Myalgic encephalomyelitis/chronic fatigue syndrome
*CPET*: Cardiopulmonary exercise testing
*VO*_*2peak*_: Peak oxygen uptake
*VO*_*2*_: Oxygen uptake
*PO*: Power output
*PPO*: Peak power output
*O*_*2*_*pulse*: Oxygen pulse
*OUES*: Oxygen uptake efficiency slope
*VE*: Minute ventilation
*PETO2*_*2*_: End-tidal partial pressure of oxygen
*PETCO2*_*2*_: End-tidal partial pressure of carbon dioxide
*VE/VO*_*2*_: Ventilatory equivalent for oxygen
*VE/VCO*_*2*_: Ventilatory equivalent for carbon dioxide
*RER*: Respiratory exchange ratio
*VT1*: First ventilatory threshold
*VT2*: Second ventilatory threshold
*RPE*: Rate of perceived exertion
*FVC*: Forced vital capacity
*FEV*_*1*_: Forced expiratory volume in 1 s
*PEF*: Peak expiratory flow
*CCC*: Canadian consensus criteria
*DSQ-PEM*: DePaul symptom questionnaire (Post-Exertional Malaise) short form
*PROM*: Patient-reported outcome measurement
