# Supplementary material for: Cardiopulmonary Exercise Testing Reveals Functional Limitations and Work Disability in Severe Post-COVID-19 and ME/CFS Patients
Source: Sports Med Open. 2026 Apr 27;12:50. doi: 10.1186/s40798-026-00995-1 (PMC13121684; doi:10.1186/s40798-026-00995-1)
Supplement: Supplementary file 1 — Supplementary Material 1. [file 40798_2026_995_MOESM1_ESM.docx]

# Supplementary Material for: Cardiopulmonary Exercise Testing Reveals Functional Limitations and Work Disability in Severe Post-COVID-19 and ME/CFS Patients

Journal: Sports Medicine – Open (Springer Nature)

Aleksandar Tomaskovic^1^, Vincent Weber^1^, David T. Ochmann^1^, Barlo Hillen^1,3^, Elmo W. I. Neuberger^1^, Alexandra Brahmer^1^, Ella Lachtermann^1^, Klaus Lieb^2^, and Perikles Simon^1^

^1^Department of sports medicine, prevention and rehabilitation, Institute for Sport Science, Johannes Gutenberg-University Mainz, Mainz, Germany

^2^Clinic of Psychiatry and Psychotherapy, University Medical Center, Johannes Gutenberg-University Mainz, Mainz, Germany

^3^Institute for Occupational, Social and Environmental Medicine, University Medical Center, Johannes Gutenberg-University Mainz, Mainz, Germany

Corresponding Author:

Prof. Perikles Simon, MD, PhD

Department of Sports Medicine, Prevention and Rehabilitation

Institute of Sport Science

Johannes Gutenberg University Mainz

Albert Schweitzer Street 22

Mainz, 55128

Germany

Phone: +49 (0) 6131 39 23 58

Email: [simonpe@uni-mainz.de](mailto:simonpe@uni-mainz.de)

**Supplementary File 1:** Absolute and relative termination criteria (Bayles et al., 2018; Fletcher et al., 2013).

| Absolute | Relative |
| --- | --- |
| Decrease in systolic BP > 10 mmHg compared to the initial BP despite an increase in stress with other signs of ischemia | Decrease in systolic BP > 10 mmHg compared to the initial BP despite an increase in stress without other signs of ischemia |
| Definite angina pectoris | Increasing angina pectoris |
| Increasing cerebral symptoms (e.g. ataxia, confusion, presyncope) | Arrhythmias other than sustained ventricular tachycardia, including multifocal ventricular extrasystoles, triplets, supraventricular tachycardia, blockages or atrial fibrillation |
| Signs of reduced peripheral perfusion (cyanosis or pallor) | Fatigue, breathlessness, wheezing, leg cramps or claudication |
| Technical reasons that make it impossible to adequately analyze the ECG or systolic BP | Development of a bundle branch block or intraventricular conduction delay that cannot be distinguished from ventricular tachycardia |
| The study participants desire to terminate the exercise | Reduced cadence < 40 rpm |
| Persistent ventricular tachycardia | Arterial hypertension (250 mmHg systolic and/or 115 mmHg diastolic) |
| ST segment elevation of at least 0.1 mV in leads without pathological Q waves (not aVR or V1) | ST or QRS changes such as horizontal or descending ST depression (> 0.2 mV) or pronounced change in position type |

BP: blood pressure; mmHg: millimeters of mercury; ECG: electrocardiogram; rpm: revolutions per minute; ST: ST segment; QRS: QRS complex; mV: millivolt; aVR: augmented vector right (ECG lead); V1: precordial lead 1 (ECG).

References:

Bayles MP, Swank AM, editors. ACSM's exercise testing and prescription. Philadelphia: Wolters Kluwer; 2018.

Fletcher GF, Ades PA, Kligfield P, Arena R, Balady GJ, Bittner VA, et al. Exercise standards for testing and training: a scientific statement from the American Heart Association. Circulation. 2013;128:873–934. doi:10.1161/CIR.0b013e31829b5b44

**Supplementary File 2:** Data processing strategies to determine VO_2peak_.

| **Reporting item** | **Description** |
| --- | --- |
| Metabolic cart | ERGOSTIK (Geratherm Respiratory, Bad Kissingen, Germany) |
| Measuring mode | breath-by-breath |
| Measuring Software | BLUE CHERRY |
| Analysis Software | R (Version 4.3.1) |
| Preprocessing | - |
| Processing strategy | low-pass forward-backward Butterworth filter with zero lag |
| Processing parameters | 3rd order, 0.04 Hz cut-off |
| Rationale | Nolte et al., 2023 |

References:

Nolte S, Rein R, Quittmann OJ. Data Processing Strategies to Determine Maximum Oxygen Uptake: A Systematic Scoping Review and Experimental Comparison with Guidelines for Reporting. Sports Med. 2023;53:2463–75. doi:10.1007/s40279-023-01903-3.

**Supplementary File 3:** Standard method (V-slope method) and Auxiliary criteria for determining first and second ventilatory threshold (VT1 and VT2) (Scharhag-Rosenberger & Schommer, 2013).

| VT1 | VT2 |
| --- | --- |
| - Field 5: VCO_2_ vs. VO_2_ (V-slope method): First significant increase in VCO_2_ compared to VO_2_ - Field 6: VE/VO_2_ and VE/VCO_2_ vs. time: Increase in VE/VO_2_ without simultaneous increase in VE/VCO_2_ - Field 9: PETO_2_ vs. time: significant increase in PETO_2_ - Field 1: VE vs. time: First significant increase in VE | - Field 4: VE vs. VCO_2_ VO_2_ (V-slope method): Significant increase in VE compared to VCO_2_ - Field 6: VE/VO_2_ and VE/VCO_2_ vs. time: Increase in VE/VCO_2_ - Field 9: PETCO_2_ vs. time: decrease in PETCO_2_ - Field 1: VE vs. time: Second significant increase in VE |

The ventilatory threshold values 1 and 2 (VT1 and VT2) are determined with the BLUE CHERRY® Diagnostic Software (Geratherm Respiratory GmbH, Bad-Kissingen, Germany) using Wassermann's 9-field graph (Kroidl et al., 2015) and the *V-slope method* as well as other auxiliary criteria (Scharhag-Rosenberger & Schommer, 2013).

References:

Kroidl RF, Schwarz S, Lehnigk B, Fritsch J. Kursbuch Spiroergometrie: Technik und Befundung verständlich gemacht. 3rd ed. Stuttgart: Thieme; 2015.

Scharhag-Rosenberger F, Schommer K. Die Spiroergometrie in der Sportmedizin. Dtsch Z Sportmed 2013. doi:10.5960/dzsm.2013.105

**Supplementary File 4:** List of all reported comorbidities (Severe comorbidities in bold).

| \| Prevalence of comorbidities \| \| \| \| \| \| --- \| --- \| --- \| --- \| --- \| \| comorbidity \| n \| prevalence \| \| severe \| \| Adipositas \| 40 \| 43.5 \| no \| \| \| Arterial hypertension \| 33 \| 35.9 \| no \| \| \| Ht hypothyreosis \| 24 \| 26.1 \| no \| \| \| Degenerative  Spine disease \| 19 \| 20.7 \| no \| \| \| Bronchial  asthma \| 15 \| 16.3 \| no \| \| \| Depressive episodes \| 15 \| 16.3 \| no \| \| \| Arthrosis \| 11 \| 12.0 \| no \| \| \| Iron deficiency \| 11 \| 12.0 \| no \| \| \| migraine \| 10 \| 10.9 \| no \| \| \| Osas \| 9 \| 9.8 \| no \| \| \| Diabetes mellitus \| 6 \| 6.5 \| no \| \| \| Anxiety disorder \| 5 \| 5.4 \| no \| \| \| Chronic Pain syndrome \| 4 \| 4.3 \| no \| \| \| Copd \| 3 \| 3.3 \| no \| \| \| Epilepsy \| 3 \| 3.3 \| no \| \| \| Mitral valve insufficiency \| 3 \| 3.3 \| no \| \| \| Osteoporosis \| 3 \| 3.3 \| no \| \| \| Polyneuropathy \| 3 \| 3.3 \| no \| \| \| Restless legs syndrome \| 3 \| 3.3 \| no \| \| \| Sleep apnea \| 3 \| 3.3 \| no \| \| \| Tricuspid insufficiency \| 3 \| 3.3 \| no \| \| \| Vitamin d deficiency \| 3 \| 3.3 \| no \| \| \| Bronchial hyperresponsiveness \| 2 \| 2.2 \| no \| \| \| **Carcinoma mamma** \| 2 \| 2.2 \| yes \| \| \| Celiac disease \| 2 \| 2.2 \| no \| \| \| Erythromelalgia \| 2 \| 2.2 \| no \| \| \| Polyarthritis \| 2 \| 2.2 \| no \| \| \| Raynaud syndrome \| 2 \| 2.2 \| no \| \| \| Recurrent Depressive disorder \| 2 \| 2.2 \| no \| \| \| Scoliosis \| 2 \| 2.2 \| no \| \| \| Sjörgen syndrome \| 2 \| 2.2 \| no \| \| \| Tonsillitis \| 2 \| 2.2 \| no \| \| \| **Acute myocarditis** \| 1 \| 1.1 \| yes \| \| \| Aortic aneurysm \| 1 \| 1.1 \| no \| \| \| Back pain snydrome \| 1 \| 1.1 \| no \| \| \| Bone tuberculosis \| 1 \| 1.1 \| no \| \| \| **Breast carcinoma** \| 1 \| 1.1 \| yes \| \| \| **Carcinoma anus** \| 1 \| 1.1 \| yes \| \| \| Cataract \| 1 \| 1.1 \| no \| \| \| Chronic bronchitis \| 1 \| 1.1 \| no \| \| \| Chronic ischemic heart disease \| 1 \| 1.1 \| no \| \| \| Chronic pansinusitis \| 1 \| 1.1 \| no \| \| | \| Prevalence of comorbidities \| \| \| \| \| --- \| --- \| --- \| --- \| \| comorbidity \| n \| prevalence \| severe \| \| Coagulation defect \| 1 \| 1.1 \| no \| \| Cochlear implantat \| 1 \| 1.1 \| no \| \| Colitis ulcerosa \| 1 \| 1.1 \| no \| \| Complex regional pain syndrome \| 1 \| 1.1 \| no \| \| Diaphragmatic hernia \| 1 \| 1.1 \| no \| \| Diastolic dysfunction \| 1 \| 1.1 \| no \| \| Diverticulitis \| 1 \| 1.1 \| no \| \| Dysesthesia \| 1 \| 1.1 \| no \| \| Exocrine pancreatic insufficiency \| 1 \| 1.1 \| no \| \| Fibromyalgia \| 1 \| 1.1 \| no \| \| Gastritis \| 1 \| 1.1 \| no \| \| Gastroenteritis colitis \| 1 \| 1.1 \| no \| \| Hepatic steatosis \| 1 \| 1.1 \| no \| \| **Hereditary spastic paraplegia** \| 1 \| 1.1 \| yes \| \| Hyperuricemia \| 1 \| 1.1 \| no \| \| Hypoxic brain injury \| 1 \| 1.1 \| no \| \| Incontinence \| 1 \| 1.1 \| no \| \| Irritable bowel syndrome \| 1 \| 1.1 \| no \| \| Maladaptation \| 1 \| 1.1 \| no \| \| **Malignant neoplasm small intestine** \| 1 \| 1.1 \| yes \| \| **Melanoma** \| 1 \| 1.1 \| yes \| \| Metabolic syndrome \| 1 \| 1.1 \| no \| \| Mitral valve prolaps \| 1 \| 1.1 \| no \| \| Myalgia \| 1 \| 1.1 \| no \| \| **Myelodysplastic syndrome** \| 1 \| 1.1 \| yes \| \| Necrotizing fasciitis \| 1 \| 1.1 \| no \| \| Nephroptosis \| 1 \| 1.1 \| no \| \| Neuralgia \| 1 \| 1.1 \| no \| \| Neurasthenia \| 1 \| 1.1 \| no \| \| Organic brain syndrome \| 1 \| 1.1 \| no \| \| Ostealgia \| 1 \| 1.1 \| no \| \| Ptsd \| 1 \| 1.1 \| no \| \| Reflux \| 1 \| 1.1 \| no \| \| Renal insufficiency \| 1 \| 1.1 \| no \| \| Respirator tract infections \| 1 \| 1.1 \| no \| \| Rupture diaphragm \| 1 \| 1.1 \| no \| \| Rupture talofibularligament \| 1 \| 1.1 \| no \| \| **Syringomyelie** \| 1 \| 1.1 \| yes \| \| Thrombosis \| 1 \| 1.1 \| no \| \| Trigeminal neuralgia \| 1 \| 1.1 \| no \| \| Varicosis \| 1 \| 1.1 \| no \| \| Ventricular extrasystole \| 1 \| 1.1 \| no \| |
| --- | --- | --- | --- | --- | --- | --- | --- | --- | --- | --- | --- | --- | --- | --- | --- | --- | --- | --- | --- | --- | --- | --- | --- | --- | --- | --- | --- | --- | --- | --- | --- | --- | --- | --- | --- | --- | --- | --- | --- | --- | --- | --- | --- | --- | --- | --- | --- | --- | --- | --- | --- | --- | --- | --- | --- | --- | --- | --- | --- | --- | --- | --- | --- | --- | --- | --- | --- | --- | --- | --- | --- | --- | --- | --- | --- | --- | --- | --- | --- | --- | --- | --- | --- | --- | --- | --- | --- | --- | --- | --- | --- | --- | --- | --- | --- | --- | --- | --- | --- | --- | --- | --- | --- | --- | --- | --- | --- | --- | --- | --- | --- | --- | --- | --- | --- | --- | --- | --- | --- | --- | --- | --- | --- | --- | --- | --- | --- | --- | --- | --- | --- | --- | --- | --- | --- | --- | --- | --- | --- | --- | --- | --- | --- | --- | --- | --- | --- | --- | --- | --- | --- | --- | --- | --- | --- | --- | --- | --- | --- | --- | --- | --- | --- | --- | --- | --- | --- | --- | --- | --- | --- | --- | --- | --- | --- | --- | --- | --- | --- | --- | --- | --- | --- | --- | --- | --- | --- | --- | --- | --- | --- | --- | --- | --- | --- | --- | --- | --- | --- | --- | --- | --- | --- | --- | --- | --- | --- | --- | --- | --- | --- | --- | --- | --- | --- | --- | --- | --- | --- | --- | --- | --- | --- | --- | --- | --- | --- | --- | --- | --- | --- | --- | --- | --- | --- | --- | --- | --- | --- | --- | --- | --- | --- | --- | --- | --- | --- | --- | --- | --- | --- | --- | --- | --- | --- | --- | --- | --- | --- | --- | --- | --- | --- | --- | --- | --- | --- | --- | --- | --- | --- | --- | --- | --- | --- | --- | --- | --- | --- | --- | --- | --- | --- | --- | --- | --- | --- | --- | --- | --- | --- | --- | --- | --- | --- | --- | --- | --- | --- | --- | --- | --- | --- | --- | --- | --- | --- | --- | --- | --- | --- | --- | --- | --- | --- | --- | --- | --- | --- | --- | --- | --- | --- | --- | --- | --- | --- | --- | --- | --- | --- | --- | --- | --- | --- | --- | --- | --- | --- | --- | --- | --- | --- | --- | --- | --- | --- | --- | --- | --- | --- | --- | --- | --- | --- | --- | --- | --- | --- | --- | --- | --- | --- | --- | --- | --- | --- | --- | --- | --- | --- | --- | --- | --- | --- | --- | --- | --- | --- | --- | --- | --- | --- | --- | --- | --- | --- | --- | --- | --- | --- | --- | --- | --- | --- | --- | --- |

*Ht hypothyreosis: hypothyroidism; Osas: obstructive sleep apnea syndrome; Copd: chronic obstructive pulmonary disease; Ptsd: post-traumatic stress disorder.*

**Supplementary File 5:** Association between Bell-Score with submaximal and maximal CPET parameters and CPET-derived Work Ability Status for the subgroup with RER ≥ 1.0.

|  |  | Bell Score |  |
| --- | --- | --- | --- |
|  | > 30 | <= 30 | **P-value** |
| **VO_2VT1_ [mL/min/kg]** | | | |
| >= 11 | 9 (28.1%) | 2 (5.9%) |  |
| < 11 | 23 (71.9%) | 32 (94.1%) | **0.036** |
| **VO_2VT1_ [%pred]** | | | |
| >= 45 | 16 (50%) | 9 (26.5%) |  |
| < 45 | 16 (50%) | 25 (73.5%) | 0.086 |
| **VO_2peak_ [mL/min/kg]** | | | |
| >= 15 | 21 (65.6%) | 9 (26.5%) |  |
| < 15 | 11 (34.4%) | 25 (73.5%) | **0.003** |
| **VO_2peak_ [%pred]** | | | |
| >= 60 | 26 (81.2%) | 20 (58.8%) |  |
| < 60 | 6 (18.8%) | 14 (41.2%) | 0.087 |
| **PPO [W/kg]** | | | |
| >= 1.0 | 22 (68.8%) | 10 (29.4%) |  |
| < 1.0 | 10 (31.2%) | 24 (70.6%) | **0.003** |
| **Work ability (CPET)** |  |  |  |
| yes | 21 (65.6%) | 7 (20.6%) |  |
| no | 11 (34.4%) | 27 (79.4%) | **< 0.001** |

*VO₂_peak_: peak oxygen uptake; PPO: peak power output; VT1: first ventilatory threshold; %pred: percent of predicted value based on age, sex, and body mass; W/kg: watts per kilogram body mass; Work ability (CPET): was classified as “no” for participants with VO₂_peak_ < 15 mL/min/kg and/or PPO < 1.0 W/kg body mass; CPET: cardiopulmonary exercise testing.*

**Supplementary File 6:** Spearman rank correlations of the Bell-Score with submaximal and maximal cardiopulmonary exercise testing parameters in all patients and stratified by Myalgic encephalomyelitis​/​chronic fatigue syndrome (ME/CFS) status and respiratory exchange ratio (RER) at exercise termination.


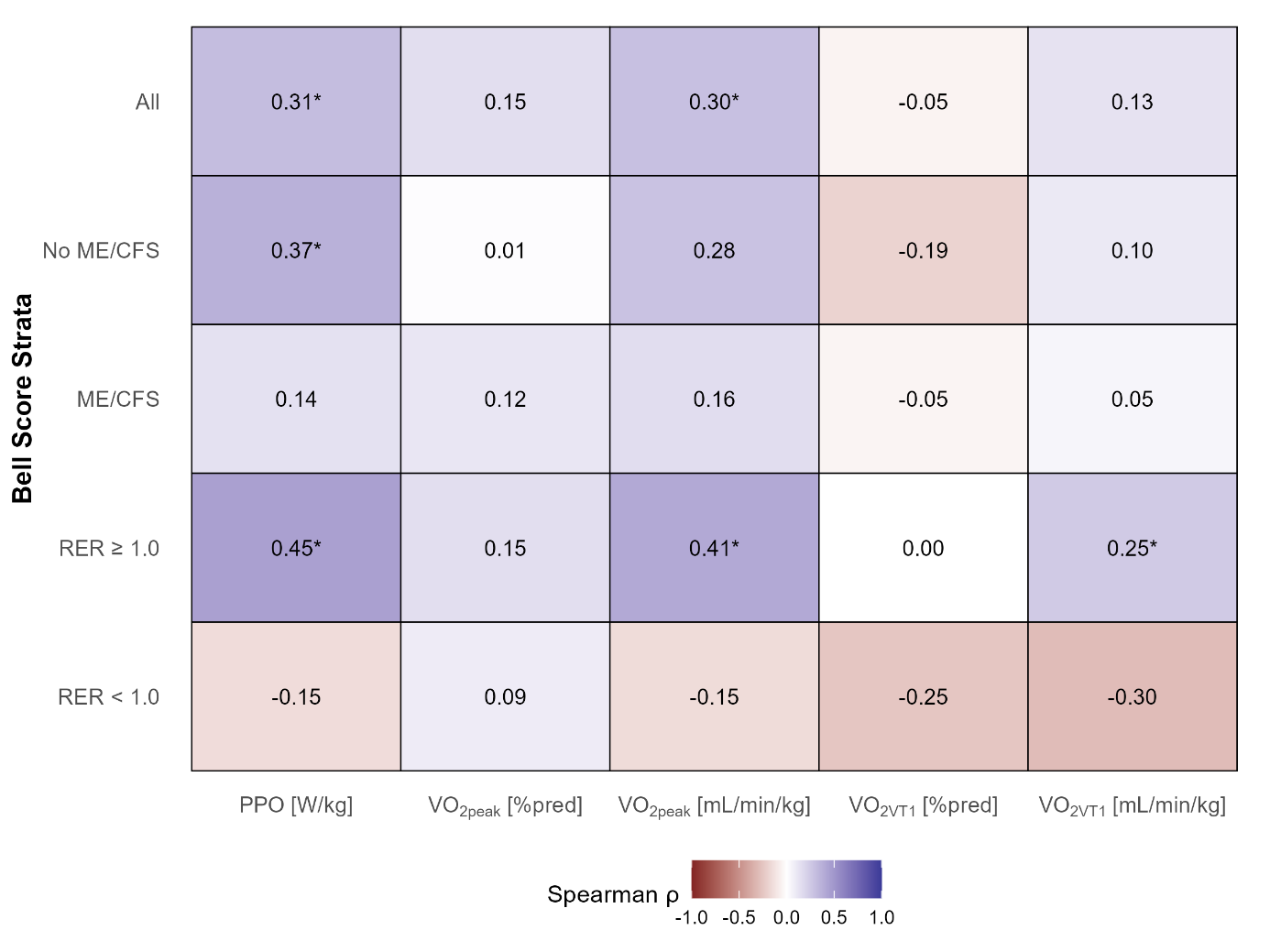


*VO_₂peak_: peak oxygen uptake; PPO: peak power output; VT: first ventilatory threshold; %pred: percent of predicted value based on age, sex, and body mass; W/kg: watts per kilogram body mass.*
